# Detecting concealed familiarity using eye movements: the role of task demands

**DOI:** 10.1186/s41235-019-0162-7

**Published:** 2019-03-29

**Authors:** Tal Nahari, Oryah Lancry-Dayan, Gershon Ben-Shakhar, Yoni Pertzov

**Affiliations:** Department of Psychology, The Hebrew University of Jerusalem, Mount Scopus Campus, 91905 Jerusalem, Israel

**Keywords:** Memory, Eye movements, Task demands, Deception, Information detection, Concealed information test, Countermeasures

## Abstract

**Background:**

What can theories regarding memory-related gaze preference contribute to the field of deception detection? While abundant research has examined the ability to detect concealed information through physiological responses, only recently has the scientific community started to explore how eye tracking can be utilized for that purpose. However, previous attempts to detect deception through eye movements have led to relatively low detection ability in comparison to physiological measures. In the current study, we demonstrate that the modulation of gaze behavior by familiarity, changes considerably when participants perform a visual detection task in comparison to a short-term memory task (that was used in a previous study). Thus, we highlight the importance of theory-based selection of task demands for improving the ability to detect concealed information using eye-movement measures.

**Results:**

During visual exploration of four faces (some familiar and some unfamiliar) gaze was allocated preferably on *familiar* faces, manifested by more fixations. However, this preference tendency vanished once participants were instructed to convey countermeasures and conceal their familiarity by deploying gaze equally to all faces. This gaze behavior during the visual detection task differed significantly from the one observed during a short-term memory task used in a previous study in which a preference towards *unfamiliar* faces was evident even when countermeasures were applied.

**Conclusions:**

Different tasks elicit different patterns of gaze behavior towards familiar and unfamiliar faces. Moreover, the ability to voluntarily control gaze behavior is tightly related to task demands. Adequate ability to control gaze was observed in the current visual detection task when memorizing the faces was not required for a successful accomplishment of the task. Thus, applied settings would benefit from a short-term memory task which is much more robust to countermeasure efforts. Beyond shedding light on theories of gaze preference, these findings provide a backbone for future research in the field of deception detection via eye movements.

**Electronic supplementary material:**

The online version of this article (10.1186/s41235-019-0162-7) contains supplementary material, which is available to authorized users.

## Significance

Research regarding the interplay between gaze behavior and familiarity provides a unique opportunity to utilize theoretical knowledge for solving real-world problems. Specifically, using eye tracking can greatly contribute to developing efficient tools for detecting concealed information, thus answering a growing need in the fields of security and law enforcement.

In two experiments, four faces were presented on a screen followed by a presentation of a single one. A dot appeared a few seconds after the onset of each display and participants were required to report its location. Some of the faces in the displays were pictures of familiar people, taken from the participants’ Facebook^TM^ accounts. In the concealed experiment, in which participants were asked to conceal the familiar faces (without specific instructions of how to do it), we found a tendency to look more at the familiar face. However, this tendency vanished in the countermeasures experiment, in which participants were asked to conceal their familiarity by deploying gaze equally to all faces. Lancry-Dayan, Nahari, Ben-Shakhar, and Pertzov (2018) used a similar design but with a short-term memory task, which yielded a preference towards unfamiliar faces that could not be completely controlled even under countermeasures. Thus, the short-term memory task is more appropriate for applied settings in which suspects might try to use countermeasure techniques to avoid detection of concealed memories. We conclude that the interaction between gaze behavior and familiarity is manifested differently in different types of tasks. Accordingly, the modulation of gaze behavior by familiarity in different tasks should be considered when designing protocols for efficient detection of concealed information in applied settings. Consequently, this study provides key principles regarding the optimal use of theory-based tasks for detecting concealed information via eye-tracking technology.

## Background

The idea of the eyes as a window to an inner world can be documented as far as the first century BC (Cicero, 1871). However, only recently has this popular concept started to gain a scientific justification. In numerous fields of research, studies have started to demonstrate that gaze behavior reflects major properties of human nature, such as personality (Hoppe, Loetscher, Morey, & Bulling, 2018), emotional state (Miltner, Krieschel, Hecht, Trippe, & Weiss, 2004; Rinck, Reinecke, Ellwart, Heuer, & Becker, 2005; Susskind et al., 2008) and cognitive process (Hayes & Henderson, 2017). Specifically, recent studies have explored how memory influences gaze behavior, and whether knowledge can be detected via eye movements. This line of research stands out by its unique combination of theoretical insights regarding memory and attention together with practical implications for forensic and security purposes.

Attempts to detect concealed information are deeply ingrained in cognitive psychology and psychophysiology research. The “Concealed Information Test” (CIT) is a theory-based method designed specifically for this purpose (e.g., Verschuere, Ben-Shakhar, & Meijer, 2011). Traditionally, physiological responses (such as heart rate and skin conductance) were recorded during a serial presentation of items, one of which is significant to a knowledgeable observer (e.g., the face of a partner in crime for a guilty suspect) but not to an unknowledgeable (innocent) one. Based on orienting response theory, the significant stimuli are likely to draw attention (Gati & Ben-Shakhar, 1990; Lykken, 1974), thus leading to different physiological responses to the significant and neutral items. Accordingly, while knowledgeable and unknowledgeable people would respond verbally in the same manner to all items (i.e., declare that they are not familiar with them), their physiological response will indicate otherwise: examinees who are familiar with a significant item are expected to exhibit a greater orienting response towards this item than to the neutral alternatives, but naïve examinees are anticipated to show similar responses to all items. Indeed, an abundance of studies has demonstrated these differential responses towards significant and neutral items, as predicted by the orienting response theory (e.g., Meijer, Selle, Elber, & Ben-Shakhar, 2014). While these studies may diverge in some characteristics (e.g., which physiological measures are recorded or the type of significant items), one of their common features is the use of the traditional serial presentation of stimuli. The adherence to this task is not surprising as the manifestation of the orienting response in physiological measures is temporally sluggish, requiring a few seconds between stimuli for the responses to return to baseline. Therefore, parallel presentation is not applicable because it is not possible to distinguish which item elicited the specific physiological response.

While physiological measures necessitate a serial presentation where each display consists of a single stimulus, eye tracking opens the field of memory detection to new possibilities by enabling a multiple-item presentation. Although some eye-tracking CIT studies used the traditional serial presentation (Millen, Hope, Hillstrom, & Vrij, 2017; Peth, Kim, & Gamer, 2013; Peth, Suchotzki, & Gamer, 2016), a handful of other studies utilized a parallel display (Schwedes & Wentura, 2012, 2016). For example, in the study of Schwedes and Wentura (2012) participants were initially familiarized with several faces, some were introduced as friends and some as foes. After a familiarization phase, participants saw a display of six faces and were instructed to identify their foes, but not to reveal their friends. Thus, if the lineup included one of their friends, they were instructed to select one of the other faces. If no known face was presented in the lineup (neutral displays), participants were instructed to select any face out of the six unknown faces.

Yet, even these studies that included a parallel presentation did not change the task demands – participants were still required to report for each item whether they were familiar with it or not. While this type of task may be suitable to the CIT based on physiological measures, it is not necessarily the best option to reveal differences in gaze behavior between familiar and unfamiliar items. Consistent with this claim, the detection efficiency when using eye tracking (whether the task included a serial or a parallel presentation) has emerged as weaker than detection efficiency based on physiological measures. This is evident from comparing the detection efficiency based on eye movements (Peth et al., 2013; Proudfoot, Jenkins, Burgoon, & Nunamaker Jr, 2016; Schwedes & Wentura, 2012) and physiological measures (Meijer et al., 2014). Moreover, a recent study by Peth et al. (2016) directly compared eye movements and physiological measures in the same study, and demonstrated a weaker detection ability of the ocular measures. However, this lower detection efficiency is not necessarily due to a shortcoming of the eye-movement measures in detecting concealed information, but might be due to a task that is not specifically tailored to reveal modulation of eye movements by familiarity.

The importance of the task in the modulation of gaze behavior has already been recognized at the onset of eye-tracking research when Yarbus (1967) showed in his pioneering study that gaze behavior changes considerably when individuals follow different tasks. Accordingly, choosing the right task may change dramatically the detection efficiency of the CIT based on eye-tracking measures. This was demonstrated in our recent study (Lancry-Dayan et al., 2018), in which we replaced the traditional task of reporting whether an item is familiar or not by a new short-memory task. In this task, after seeing a display of four faces, participants saw a single face and were asked to decide whether this face appeared in the previous display. Obviously, one of the advantages of this task is the combination of a simultaneous presentation of items together with the traditional single presentation. However, this task has an additional imperative advantage – it is based on solid theoretical foundations regarding the expected differences in gaze behavior towards familiar and unfamiliar items. On the one hand, since familiar objects are expected to require fewer resources during encoding into memory (Jackson & Raymond, 2008), they should attract less attention than unfamiliar items during the parallel display. This difference should manifest in eye movements, and would result in more direct fixations on unfamiliar faces. On the other hand, based on previous findings (Gati & Ben-Shakhar, 1990), we anticipated an orienting response towards the familiar face, resulting in more direct viewing time at the beginning of the display. This theory-based task indeed bears fruit; we found that viewing time during the parallel display was characterized by two phases, starting with a preference towards the familiar face followed by continuous avoidance. Interestingly, the strong avoidance was evident even when participants were explicitly instructed to conceal their familiarity by deploying their gaze equally to all faces. By exploiting these patterns, a machine learning classification algorithm and signal detection analysis revealed impressive detection efficiency estimates (over 88% classified correctly by the support vector machine – SVM; an area under the Receiver Operating Characteristic (ROC) curve of at least .89), higher than other studies that used physiological, behavioral or eye-tracking measures.

In the above study, all three experiments employed the same short-term memory task. Therefore, it is not clear whether the pattern of preference and avoidance is due to the specific short-term task or reflects a more general gaze tendency towards familiar faces. Understanding the link between task demands and the influence of memory on gaze behavior will broaden the theoretical framework of memory-guided attention, and will allow for designing better paradigms and tools for detecting concealed information. In our current study, we set to explore the role of task and instructions on the modulation of ocular behavior by familiarity. Specifically, we ran two experiments that are identical to the experiments reported by Lancry-Dayan et al. (2018) in terms of their visual input, but differ in terms of their task demands. Specifically, the current study employs a visual detection, rather than a short-term memory task. In other words, the sole difference between the previous short-term memory and the current visual detection tasks is in the task demands. While, in the previous task, participants were asked to report whether a single face had appeared in the previous simultaneous display or not, in the current task participants only had to report the spatial location of a gray dot that appeared on a random location on the stimuli. If indeed the short-term memory task modulated the gaze behavior towards the familiar faces, we anticipate that the change of task will change the pattern of gaze behavior. We hypothesize that the avoidance effect depends mostly on the short-term memory task, in which there is an advantage in fixating more on the unfamiliar faces during encoding. However, this advantage might have masked the orienting response-related preference tendency towards the familiar face. As the new task does not require encoding at all, and the familiar items should still elicit an orienting of attention, we hypothesize that it will allow for a greater manifestation of the preference towards familiar faces. The fact that different tasks elicit different patterns of gaze behavior is highly important for concealed memory detection paradigms. If a specific task enhances the differential gaze characteristics towards familiar and unfamiliar stimuli, this task will also enable better detection ability. While previous studies mainly used the same experimental paradigm and examined different physiological measures (from reaction time to electroencephalogram (EEG)-event -related potentials), we changed the experimental paradigm and examined how it affects familiarity-related ocular measures, trying to find the optimal task for concealed information detection.

Moreover, we intend to examine the extent to which gaze behavior can be controlled by explicit instructions regarding how to deploy gaze. Understanding whether gaze behavior is controllable or not will not only shed light on the ability to control gaze according to high-level goals, but is also important from an applied perspective. Specifically, in real-life settings guilty examinees may try to conceal their information by actively applying various methods to fool the test (i.e., countermeasures). Several studies have demonstrated that countermeasures can significantly attenuate the detection efficiency of the CIT (see a review in Ben-Shakhar, 2011). Accordingly, it is important to establish the resilience of the eye-tracking-based CIT to countermeasures by developing efficient methods that are not prone to such manipulations.

## Methods

**Fig. 1:**
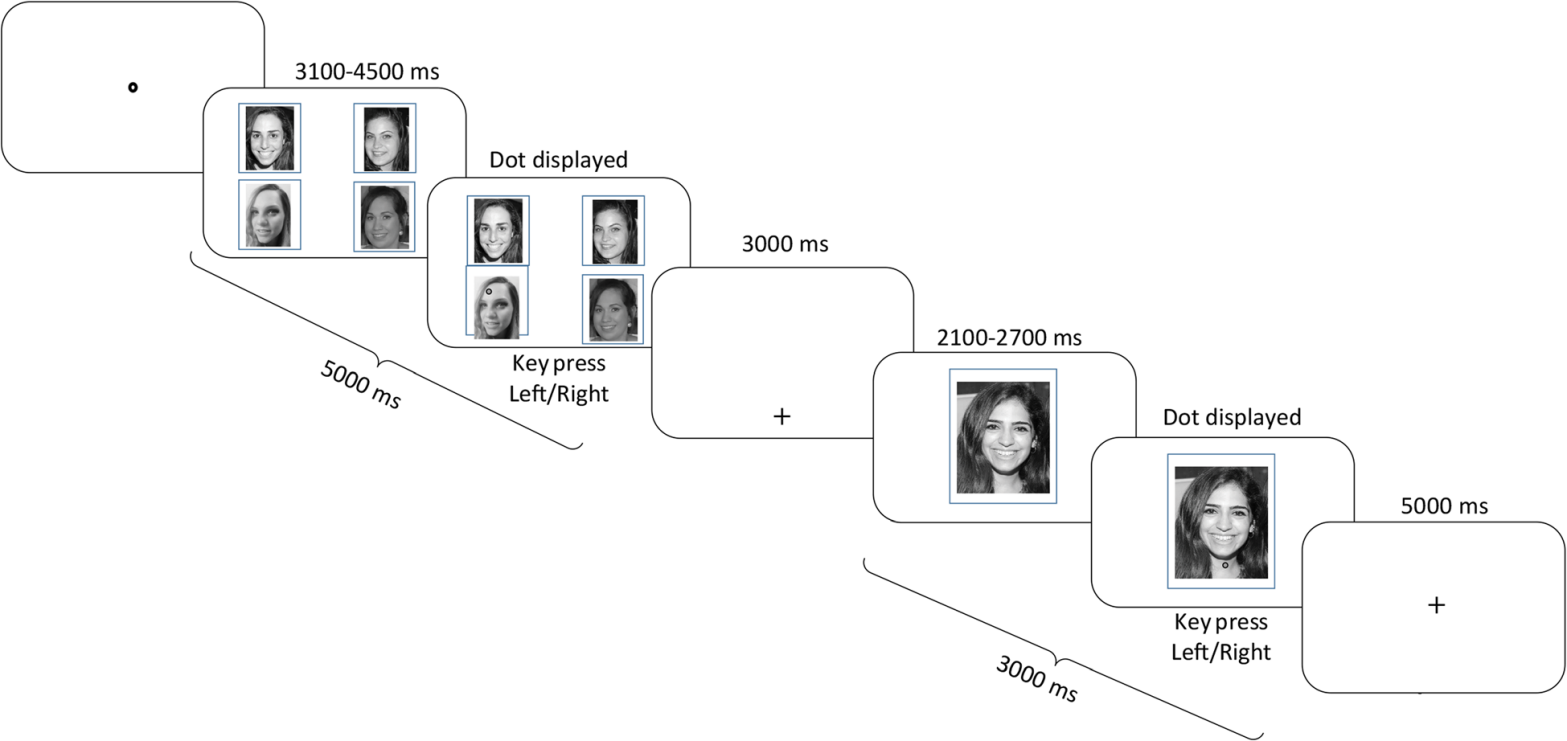
Illustration of the experimental procedure. A trial begins with a parallel display of four faces. A gray dot appears on one of the faces, between 3100 and 4500 ms after the parallel display onset, and participants are asked to report whether it appeared on the right or left side of the screen. Afterwards, a blank screen is presented followed by a single face. Between 2100 and 2700 ms after the single display onset, a gray dot appears on the face and participants are required to report again on which side of the screen the dot apeared. A personally familiar face could be presented in the parallel display, in the single display, in both displays or in none of them

We designed a new detection paradigm with a similar visual input to the one used by Lancry-Dayan et al. (2018). Similarly to the previous short-term memory task, in this visual detection task four faces were presented on the screen followed, after a short delay, by a presentation of a single face. During each of the two displays, participants were required to report by a key press when they detected a gray dot that emerged a few seconds after the display onset (see the “Procedure” section and Fig. 1). The task ensured that participants will look at all faces, but the familiarity of the faces was orthogonal to the task. Accordingly, unlike the short-term memory task used in our previous study (Lancry-Dayan et al., 2018), there was no advantage in looking more on unfamiliar faces.

This task was carried out in two experiments, which differed only in the concealment instructions. Participants in the first experiment were instructed to perform the visual detection task and “conceal their familiarity with the faces,” without explicitly explaining how to conceal (referenced as “concealed”). In the second experiment, concise instructions regarding how to conceal familiarity were included: “In order to conceal your familiarity with the pictures, try to look at all the faces equally” (referenced as “countermeasures”). Beyond assessing the detection efficiency of the paradigm, this design allowed us to investigate the temporal dynamics of gaze allocation during a visual detection task and to explore the influence of task and long-term memory on gaze behavior, as well as how it can be modulated by intentional control.

### Participants and apparatus

We tried to reach the same sample size used in Lancry-Dayan et al. (2018). After exclusion of some participants (see below), the concealed experiment included 33 participants (seven males), and the countermeasures included 28 participants (eight males) ranging in age from 19 to 28 years. All participants had normal or corrected-to-normal vision. In both experiments, some participants registered for the experiments but did not show up (concealed, 5/38; countermeasures, 7/37). In the countermeasures experiment, two additional participants were excluded due to technical problems.

### Stimuli

Similar to the previous study, we used photos of friends of the participants taken from their Facebook™ account. Prior to the experiment, participants provided the names of eight women and eight men they knew, and rated their familiarity with each person on a scale from 1 (we see each other approximately twice a year or less) to 5 (we see each other on a daily basis). For each participant, four pictures of women and four of men were taken with permission from public pictures on Facebook™. Pictures with a straightforward head position were selected, transformed into black and white images, and normalized to have similar average brightness using Matlab (MATLAB 8.6, The MathWorks Inc., Natick, MA, USA, 2015).

In order to provide full counterbalancing, the familiar faces of each participant were the other participants’ unfamiliar faces. To do so, pictures of eight participants were grouped together to generate the stimulus sets for the parallel display, resulting in a total of 64 pictures in each set, with eight familiar faces and 56 unfamiliar faces for each participant (the unfamiliar faces consisted of the eight familiar faces of the other seven participants in the same group). For the single display, the pictures of four participants were grouped together, resulting in a total of 32 pictures in each set, repeated twice during the experiment (64 trials in total). The 32 pictures consisted of eight familiar faces and 24 unfamiliar faces for each participant (the unfamiliar faces consisted of the eight familiar faces of the other three participants in the same group). Accordingly, the eight participants in each group saw the same parallel displays with half of them also seeing the same single displays.

The stimuli were displayed on a 23″ Syncmaster monitor, with a 120-Hz refresh rate, and a 1024 × 768 screen resolution. Monocular gaze position was tracked at 1000 Hz with an EyeLink 1000+ (SR Research Ltd., Mississauga, ON, Canada). Participants’ heads were stabilized using a chinrest, situated 60 cm from the screen.

### Procedure

At the beginning of the experiment, each participant went through the standard 9-point calibration and validation procedure provided with the eye tracker. Each participant completed at least three correct practice trials out of five. Participants who failed more than two out of the five trials underwent another session of five training trials. The practice session was designed to train the participants to adequately perform the visual detection task and used a different set of faces taken from Facebook^TM^, all of them unfamiliar to participants. Thus, none of the stimuli in the practice sessions were used later in the experiment. Each one of the 64 trials started with a fixation validation process, allowing a deviation of only 1° of visual angle between the predicted gaze position and the center of the fixation point. Larger deviations were accompanied by an error beep and led to a repeated calibration process. Fixation validation was followed by a parallel display of four faces (5000 ms), followed by a blank fixation interval (3000 ms), a single-face display (3000 ms) and a blank screen with a central fixation point (5000 ms). During the parallel display a dot appeared after a random period, between 3100 and 4500 ms after the initial presentation of the faces. In the single display, the onset of the dot was 2100–2700 ms after the single face appeared. The fixation point prior to the single display was displayed below the face in order to refrain from biasing gaze position to any specific location on the face (Arizpe, Kravitz, Yovel, & Baker, 2012; Peterson & Eckstein, 2013).

During both displays, participants were required to report whether the dot appeared on the left or the right side of the screen (key press: left/right). The main purpose of this assignment was to make sure that participants are scanning the faces. In the parallel display, the dot’s location was randomly assigned to one of the four faces and its position on the face was randomly determined out of all possible locations on the face area. In the single display, the dot appeared on a random position on the face area. The dot emerged several seconds after both displays’ onset in order to provide a few seconds of “clean” gaze behavior before detection happened. In the single display, detection reports were enabled throughout the display and during the white screen that followed, in order to provide ample time to react (see Fig. 1).

A familiar face could appear on the parallel display, the single display, both displays, and none of the displays. In the parallel display, half of the trials consisted of only unfamiliar faces and the other half consisted of one familiar and three unfamiliar faces. Accordingly, each familiar face appeared in four trials, once in each location of the parallel display (top right, top left, bottom right, bottom left). In the single display, each familiar face appeared twice. Because the familiar faces of one participant were the unfamiliar faces of another participant, all faces appeared once in each location in the parallel display and twice in the single display. This design ensured that all faces were completely counterbalanced, such that each face appeared equally in all possible locations and displays.

### Data acquisition and exclusion criteria

During debriefing, all participants reported whether the 64 pictures in their dataset were familiar to them or not. Pictures were discarded from further analysis if participants marked them as familiar on the pre-experiment questionnaire but did not recognize them during the debriefing, or if they were recognized during debriefing but were not included in the list of familiar faces supplied in the pre-experiment questionnaire (overall 2.7% and 3.0% of the pictures in the concealed and countermeasures experiments, respectively).

Eye-movement data were parsed into saccades and fixations using EyeLink’s standard parser configuration: samples were defined as a saccade when the deviation of consecutive samples exceeded 30°/s velocity or 8000°/s^2^ acceleration. Samples gathered from time intervals between saccades were defined as fixations.

### Data analysis

The ocular measures in the parallel and single displays were extracted only for the period of time before the appearance of the dot. (i.e., for both displays, we took the shortest time interval without the dot). Therefore, the analyses below refer to the first 3000 ms and first 2000 ms of the parallel and single displays, respectively.

Based on our previous study (Lancry-Dayan et al., 2018) and others (Ryan, Hannula, & Cohen, 2007), for each parallel display we extracted the gaze dwell time during the first 3000 ms of the trial. Overall gaze dwell time on an area can be parceled according to both the number of times that this area was visited and the number of fixations directed to that area. Accordingly, we examined the number of times gaze entered each face (visits) and the number of fixations on each face. As overall gaze time is not only related to the number of visits and fixations, but also to their duration, in the current study we additionally analyzed the duration of visits and fixations. Finally, the measures extracted during the single display were the mean fixation duration, reaction time and accuracy of the detection task. For elaboration on all ocular measures, see Table 1.

**Table 1:** Definition of the different ocular measures used in the statistical analysis

	Measure	Description
Parallel display	Dwell time	Total time (in ms) that gaze was directed to an area during the first 3000 ms of the trial
	First and second dwell-time intervals	Dwell time during the period before the dot appearance was divided into two: first interval of the trial (0–1000 ms) and second interval of the trial (1000–3000 ms)
	Number of visits	Number of times each face area was visited during the first 3000 ms of the trial. A single visit consists of all consecutive fixations on a specific area before moving out of that area
	Duration of visits	The mean duration (in ms) of visits for each face (during the first 3000 ms of the trial)
	Number of fixations	Number of fixations on a face (during the first 3000 ms of the trial)
	Duration of fixations	The mean duration (in ms) of fixations on a face (during the first 3000 ms of the trial)
Single display	Mean duration of fixation	The mean duration (in ms) of fixations directed to the single-face (during the first 2000 ms of the trial)

#### Time course analysis

To assess the gaze position dynamics throughout the parallel display, we performed a time course analysis of the proportion of time fixation was directed to familiar vs. unfamiliar faces, similar to Lancry-Dayan et al. (2018). Each trial that included a familiar face was parceled into 100-ms bins. In each time bin we calculated the proportion of time that gaze was directed to familiar faces, unfamiliar faces, or outside of any interest area/blinks. Dwell time on the three unfamiliar faces was pooled and divided by three to make it comparable to the dwell time on the familiar face. For each time bin we contrasted the proportion of fixation time on the familiar and unfamiliar faces and applied a correction for multiple comparisons using the False Discovery Rate (FDR; Benjamini & Hochberg, 1995).

#### Visit analysis

For each parallel display that included a familiar face, we analyzed the number of times that each face was visited and the mean duration of each visit (during the first 3000 ms of the display). A visit is defined as consecutive fixations within the same face, before a saccade is made outside of it. For the three unfamiliar faces, we calculated the mean number of visits by summing the number of visits for all the unfamiliar faces and dividing it by three. For the mean visit duration calculation, we took the dwell time on the familiar and the overall dwell time on the unfamiliar faces separately, and divided it by the number of visits. Finally, we averaged, for each participant, the number of visits and their duration across all trials, separately for familiar and unfamiliar faces. This analysis of visits is slightly different from the analysis that was carried out in our previous study (Lancry-Dayan, Nahari, Ben-Shakhar, & Pertzov, 2018). See additional file 1 for elaboration.  

#### Fixations analysis

For each parallel display that included a familiar face, we analyzed the number of fixations that were directed to each face and their mean duration (during the first 3000 ms of the display). The analysis was carried out in an identical manner to the visit analysis. For the three unfamiliar faces, the mean number of fixations was achieved by summing the number of fixations on all the unfamiliar faces and dividing it by three. For the mean fixation duration, we took the dwell time on the familiar and the overall dwell time on the unfamiliar faces separately, and divided it by the number of fixations. Finally, we averaged, for each participant, the number of fixations and their duration across all trials, separately for familiar and unfamiliar faces.

#### Bayesian modeling

We used two additional Bayesian approaches to analyze our data: parameter estimation and model comparison. For both analyses, we calculated the expected value of the effect size, using Cohen’s *d* (Cohen, 1992), that captures the difference between the within-subject expected value of familiar and unfamiliar faces divided by the standard deviation (Rouder, Speckman, Sun, Morey & Iverson, 2009). For estimation purposes, we calculated the high-density interval (HDI) of the posterior distribution of each parameter.

Bayes factor (BF) was calculated based on the Savage-Dickey density ratio method (Wagenmakers, Lodewyckx, Kuriyal & Grasman 2010). The null model suggests no difference in behavior between the familiar and unfamiliar faces in each measure and accordingly the expected effect size should be zero. The alternative model suggests that the effect size, delta (*δ*), is different from zero. Dividing the height of the posterior distribution of *δ* by the height of the prior distribution of delta, at the point of interest, which in this case is zero, provides the Bayes factor.

As suggested by Kruschke (2014) and Lee and Wagenmakers (2013), in order to prevent biasing of the parameters, we used non-informative priors, based on the standard deviation of the pooled data (familiar and unfamiliar faces). Accordingly, we estimated the expected value of the difference between familiar and unfamiliar faces using a normal distribution centered around the effect size × sigma, with a standard deviation estimated from a uniform distribution ranging from 0 to 10 times the standard deviation of the pooled data (*μ*_*differences*_~ *Normal*(*δ* × *σ*, *σ*)) *μ*_*unfamiliar*_~*Normal*(*mean*_*pooled*_, 10 × *S*_*pooled*_), whereas *σ*~*uniform*(0, 10 × *sd*), assuring a wide enough range of the prior distribution. For the estimation of the Cohen’s *d* we used a Cauchy prior (*δ*~*Cauchy*(0, 1)). This prior distribution is centered with high density around zero (in accordance to the null model) and is traditionally used for effect size estimation (Rouder et al., 2009). See Additional file 1 for the model and the exact priors for the analysis.

#### Receiver Operating Characteristic (ROC) curves

From an applied perspective it is important to assess the detection efficiency of the eye-tracking measures in differentiating between knowledgeable (guilty) and non-knowledgeable (innocent) individuals. CIT detection efficiency has traditionally been evaluated by signal detection measures such as ROC curves and the areas under the curves (e.g., Ben-Shakhar, Lieblich, & Kugelmass, 1970; National Research Council, 2003). The area under the curve describes the detection efficiency of the CIT and varies from 0 to 1, with a chance level of 0.5 (for a more detailed description of signal detection analysis as applied to detection of concealed information, see Lieblich, Kugelmass, & Ben-Shakhar, 1970). As no sample of non-knowledgeable participants (for whom all the faces were unfamiliar) was included in this study, we used the trials that contained no familiar faces for that purpose. Such trials basically simulate a non-knowledgeable (innocent) sample that has no familiarity with any of the pictures. Accordingly, eight unfamiliar faces that appeared in the displays that contained only unfamiliar faces were selected for each participant. These faces were the familiar faces of another participant, thus ensuring that the sole difference between the two sets was the personal familiarity with the face. In other words, for each participant, one set contained eight familiar faces, and the other contained eight unfamiliar faces that were the familiar faces of another participant.

In order to construct the ROC curves and compute the areas under the curves, we used the four measures extracted from the parallel display: dwell time before the dot appeared divided into two phases (0–1000, 1000–3000 ms)^1^, number of visits and total fixation count (see Table 1), and the measures from the single display: mean fixation duration and reaction time in the detection task^2^. To combine all the different measures (despite the differences in units), we standardized each measure within each participant across all stimuli (Ben-Shakhar, 1985). The Z scores of the familiar faces were based only on displays that contained a familiar face, while the Z scores of the unfamiliar faces (simulating unknowledgeable participants) were based on displays that contained only unfamiliar faces. Then, we created a single detection score by averaging the six different Z scores, corresponding to the six different measures, using a conservative method of equal weights. Finally, the detection score distribution of the familiar faces was compared to the detection score distribution of the unfamiliar faces to create an ROC curve.

## Results

### Parallel four-face display

**Fig. 2:**
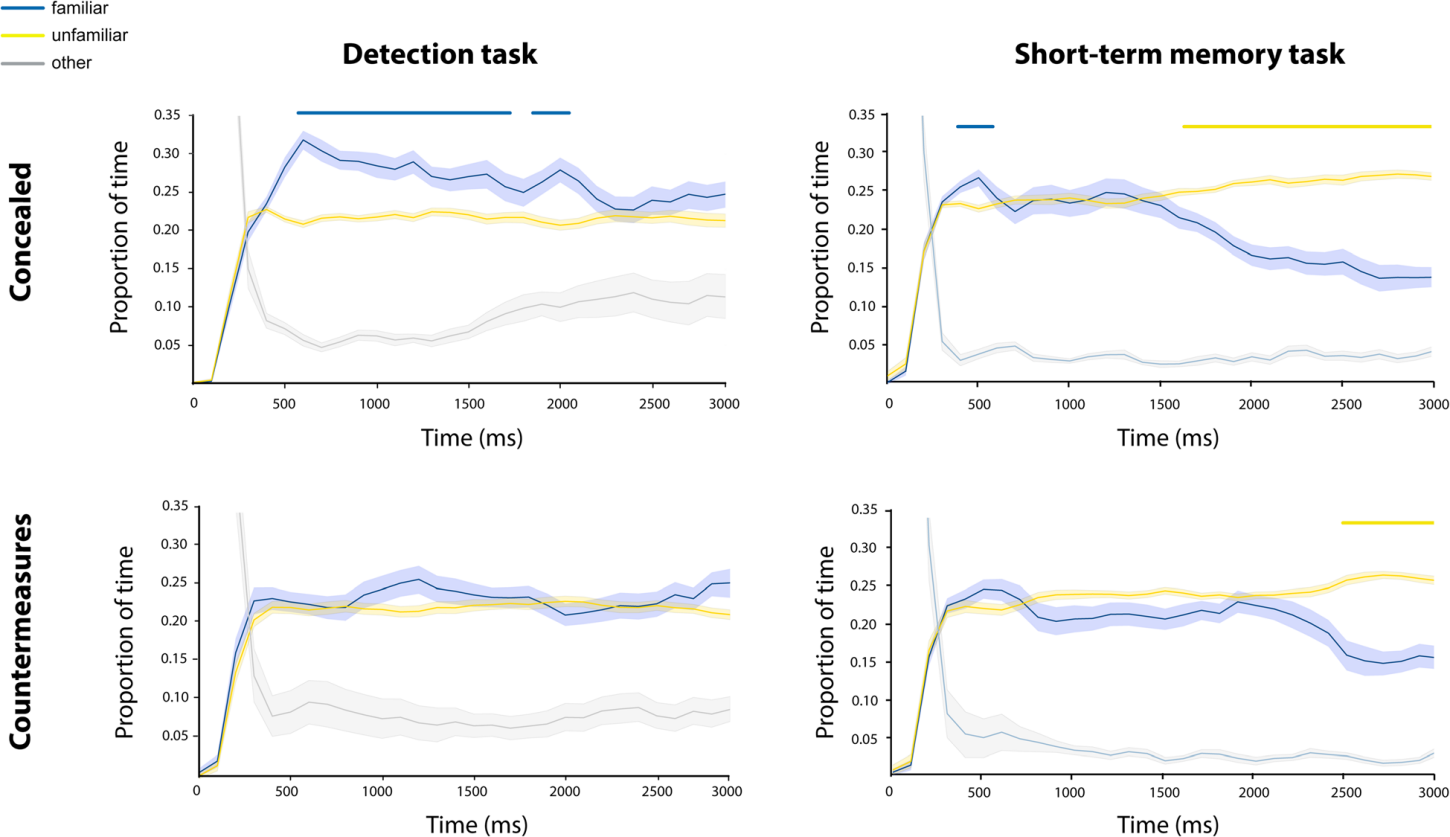
Time course of gaze position in the current study (left panel) and in the previous one (right panel). The concealed experiments are displayed on top and the countermeasures at the bottom. The graph depicts the proportion of fixation time directed at familiar (blue) and unfamiliar (yellow) faces (averaged across the 3 unfamiliar faces) during the parallel display. Eye movements outside the interest areas or blinks are presented in gray. Time points with a significant difference (after False Discovery Rate (FDR) correction) between familiar and unfamiliar faces are displayed at the top of the figure as blue or yellow bars for familiarity preference and avoidance effects, respectively. Shadowed area indicates ± 1 standard error (SE) across participants

As hypothesized, the pattern of gaze in both experiments was different from the pattern observed in the short-term memory task (see Fig. 2). In the concealed experiment, a stable preference towards the familiar face was observed from around 500 ms after display onset until about 2200 ms (significant below α < .05, except of a small gap of 200 ms, after FDR correction for multiple comparisons). In contrast, in the countermeasures experiment (in which participants were explicitly instructed to deploy gaze equally at all faces) we did not find a statistically significant preference effect.

**Fig. 3:**
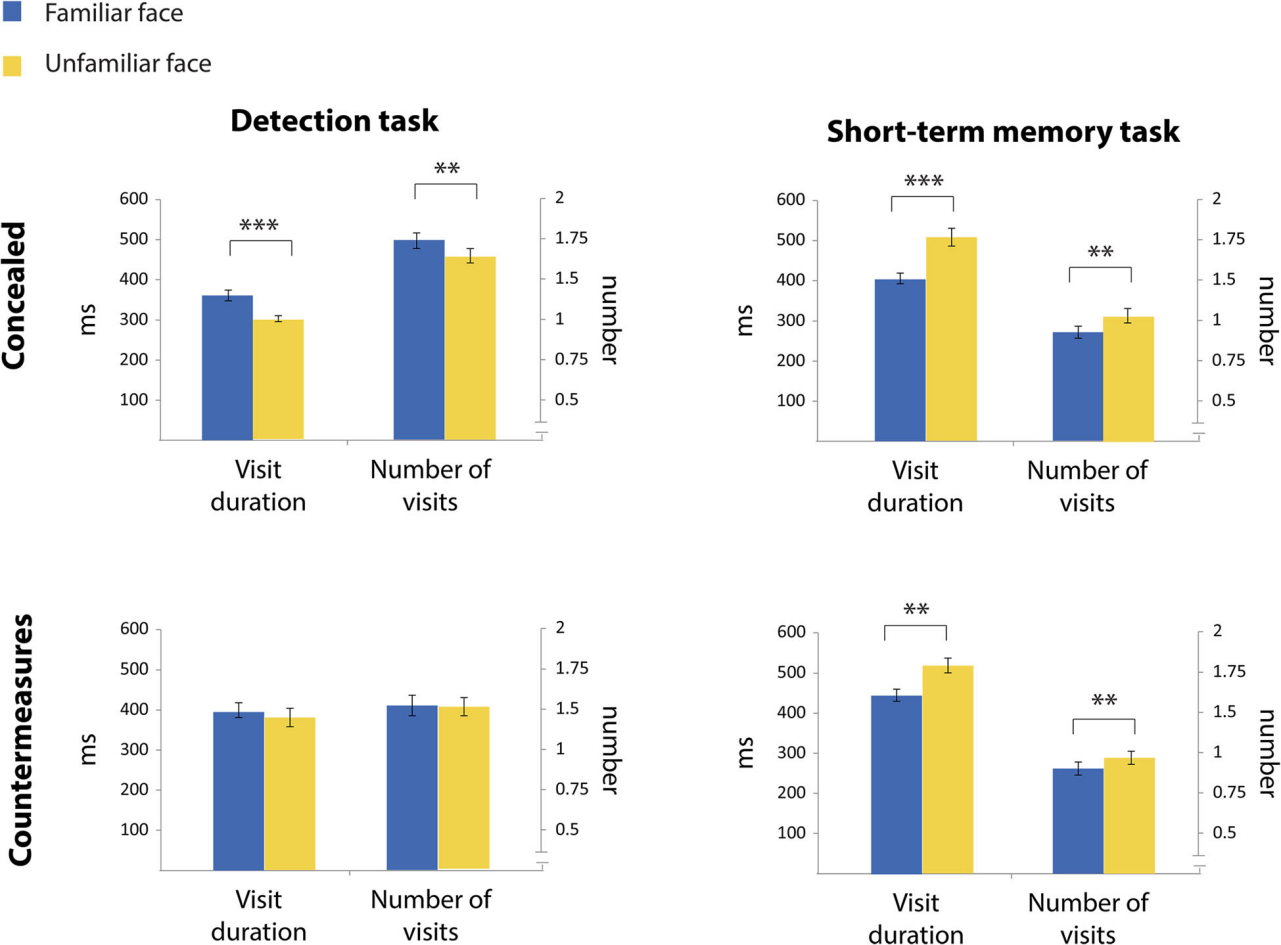
Number of visits and durations in the current study (left panel) and in the previous one (right panel). Each graph depicts the mean duration of visits (consecutive fixations on a specific face before moving to a different face) and their number, separately for familiar (blue) and unfamiliar (yellow) faces. Error bars indicate ± 1 standard error (SE). (**p value < 0.01, ****p value < 0.001 for paired *t* test comparisons between visits to familiar and unfamiliar faces)

The overall direct gaze duration on an area is a product of the number of times that gaze visited this area multiplied by the average time that it spent there on each visit. Accordingly, we compared the number of visits and their duration on familiar faces and unfamiliar faces. In the concealed experiment, familiar faces were visited more times (*t* (32) = 5.675, *p* < .001, *d* = 0.99) and for longer durations (*t*(32) = 3.688, *p* = .001, *d* = 0.64) than unfamiliar faces. In contrast, in the countermeasures experiment there were no significant differences between familiar and unfamiliar faces regarding both the number of visits (*t*(27) = 0.905, *p* = .374, *d* = 0.17) and their duration (*t*(27) = 0.298, *p* = .768, *d* = 0.06). For comparison with the results of the short-term memory task, see Fig. 3.

The Bayesian analysis yielded a similar pattern of results. In the concealed experiment, the alternative model of the visits duration was favored over the null model (BF_10_ = 1923.91, estimated *d* = 0.94, HDI [0.53, 1.35]), as well as the alternative model of the number of visits (BF_10_ = 27.36, estimated *d* = 0.6, HDI [0.24, 0.98]). As expected, a strong support for the null model was found in the countermeasures experiment (number of visits: BF_10_ = 0.15, estimated *d* = 0.05, HDI [− 0.31, 0.4]; visit duration: BF_10_ = 0.21, estimated *d* = 0.16, HDI [− 0.21, 0.52]).

**Fig. 4:**
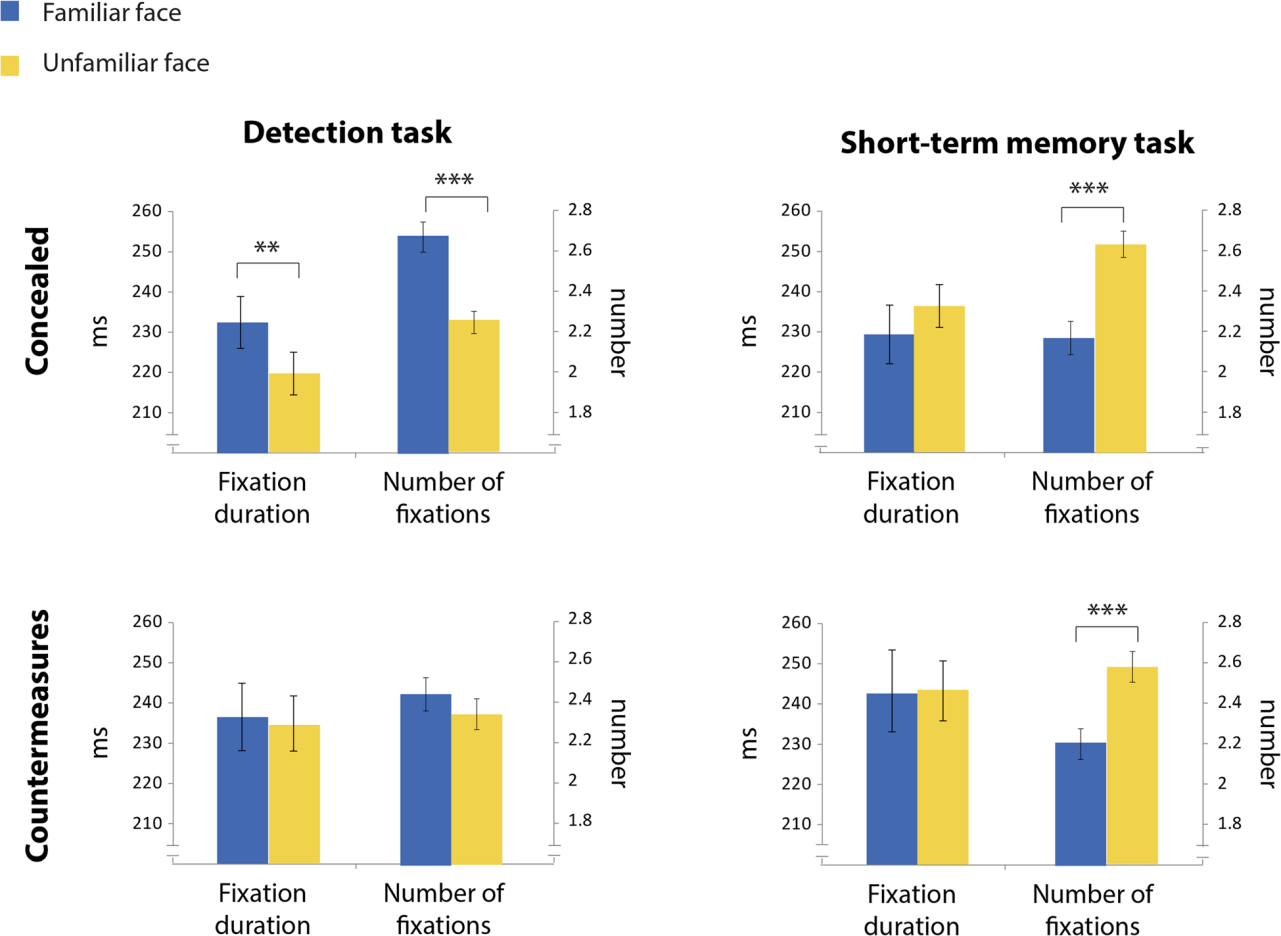
Fixation analysis in the current study (left panel) and in the previous one (right panel). Each graph depicts the mean duration of fixations and their number, separately for familiar (blue) and unfamiliar (yellow) faces. Error bars indicate ± 1 standard error (SE). (**p value < 0.01, ****p value < 0.001 for paired *t* test comparisons between visits to familiar and unfamiliar faces)

In addition, overall direct gaze duration on an area is also a product of the number of fixations directed to that area and their average duration. Accordingly, the gaze patterns that are described above can be investigated also by examining the number of fixations on familiar and unfamiliar faces and their mean duration. This analysis yielded similar results to the visit analysis; while in the concealed experiment participants executed more fixations toward familiar faces (*t*(32) = 6.095, *p* < .001, *d* = 1.06) and for longer duration (*t*(32) = 3.056, *p* = .005, *d* = 0.53), no significant differences in the number of fixations (*t*(27) = 1.442, *p* = 0.161, *d* = 0.27) or their duration (*t*(27) = 0.408, *p* = .686, *d* = 0.07) were evident in the countermeasures experiment. The results of this analysis, along with the results of the previous short-term memory task study are depicted in Fig. 4.

These results were supported also by the Bayesian analysis which favored the alternative model in the concealed experiment for the number of fixations (BF_10_ = 4760.11, estimated *d* = 1.0, HDI [0.59, 1.45]), as well as for the fixations duration (BF_10_ = 6.43, estimated *d* = 0.5, HDI [0.15, 0.86]). However, in the countermeasures experiment the null model was favored over the alternative one, for both measures (number of fixations: BF_10_ = 0.36, estimated *d* = 0.25, HDI [− 0.11, 0.62]; duration of fixations: BF_10_ = 0.15, estimated *d* = 0.07, HDI [− 0.29, 0.42]).

### Single-face display

In contrast to previous findings (Althoff & Cohen, 1999; Heisz & Shore, 2008) and to the short-term memory task, there was no significant difference in the mean duration of fixations directed to familiar and unfamiliar faces during the single display, for both the concealed experiment (*t*(32) = 1.87, *p* = 0.07, *d* = 0.33) and the countermeasures experiment (*t*(27) = .994, *p* = .329, *d* = 0.18). Moreover, the Bayesian analysis favored the null model over the alternative, for both the concealed experiment (BF_10_ = 0.66, estimated *d* = 0.31, HDI [− 0.03, 0.65]) and the countermeasures experiment (BF_10_ = 0.23, estimated *d* = 0.17, HDI [− 0.17, 0.52]).

**Fig. 5:**
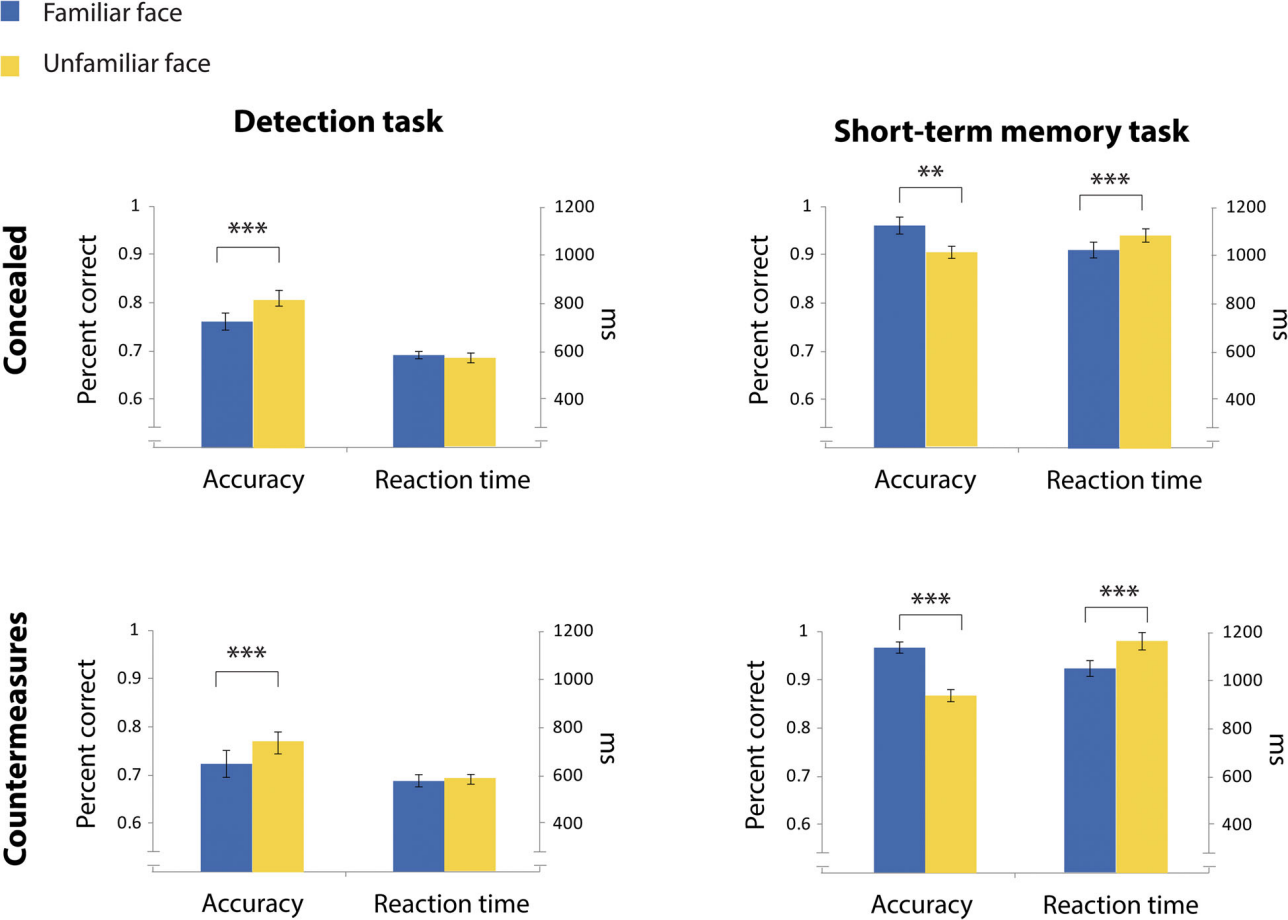
Accuracy and reaction time in the detection task (left panel) and in the short-term memory task (right panel) during the single-face display. Error bars indicate ± standard error (SE). (*p value < 0.05, **p value < 0.01, ***p value < 0.001 for paired *t* test comparisons between familiar and unfamiliar faces)

As in the short-term memory task, we examined the reaction time and accuracy of the dot detection task in the single display (see Fig. 5). The results were similar in both experiments: mean reaction time was similar when familiar and unfamiliar faces were displayed (concealed: *t*(32) = 1.29, *p* = 0.10, *d* = 0.22; countermeasures: *t*(27) = 0.588, *p* = 0.28, *d* = 0.11), participants were significantly less accurate when the dot appeared on a familiar face (concealed: *t*(32) = 4.24, *p* < .001, *d* = 0.74; countermeasures: *t*(27) = 3.51, *p* < .001, *d* = 0.66). The Bayesian analysis demonstrated a higher probability for the null model regarding the reaction time in both the concealed (BF_10_ = 0.29, estimated *d* = 0.21, HDI [− 0.21, 0.54]) and countermeasures experiments (BF_10_ = 0.17, estimated *d* = 0.1, HDI [0.25, 0.46]). In contrast, a higher probability of the alternative model of the accuracy measure was obtained for both the concealed (BF_10_ = 95.0, estimated *d* = 0.7, HDI [0.3, 1.07]) and countermeasures (BF_10_ = 17.67, estimated *d* = 0.62, HDI [0.22, 1.03]) experiments.

### Direct comparison between studies – parallel display

To further understand how the task influenced familiarity effects on gaze behavior, we conducted a four-way analysis of variance (ANOVA) on the dwell time during the parallel display, with two within-subject factors (familiarity: familiar vs. unfamiliar and dwell time phase: first vs. second) and two between-subject factors (type of task in the different studies: short-term memory task vs. visual detection task and experiment instructions: concealed vs. countermeasures). As the main interest of this analysis is to investigate the effect of familiarity in the different studies, we discuss here only the effects that include these two factors (three-way interactions and below). For the complete pattern of results, see Additional file 1: Table S1.

As expected, we found a significant triple interaction between familiarity, task and dwell time phase [*F*(1,115) = 92.065, *p* < .001, *η*^2^_p_ = .445], indicating that the two tasks elicit different modulation of gaze behavior by familiarity in the two dwell time phases. To explore how this interaction between familiarity and dwell time phase differed between the two tasks, we conducted a two-way ANOVA for each task with two within-subject factors (familiarity: familiar vs. unfamiliar and dwell time phase: first vs. second). This analysis revealed a significant interaction effect in both tasks (short-term memory task: *F*(1,57) = 82.429, *p* < .001, *η*^2^_p_ = .591; detection task: *F*(1,60) = 4.679, *p* = .035, *η*^2^_p_ = .072). Thus, the triple interaction reflects differences in the sizes of these two-way interactions, demonstrating a much stronger interaction between phase and familiarity in the short-term memory task.

Moreover, there was also a significant triple interaction between familiarity, task and experiment instructions [*F*(1,115) = 7.615, *p* = .007, *η*^2^_p_ = .062]. This interaction reflects a difference between the two tasks in the modulation of gaze behavior by familiarity in the concealed and countermeasures experiments. To further understand this difference between the two tasks, we carried out a two-way ANOVA for each task with one within-subject factor (familiarity: familiar vs. unfamiliar) and one between-subject factor (experiment instructions: concealed vs. countermeasures). While there was a significant interaction between familiarity and experiment instructions in the visual detection task [*F*(1,59) = 8.264, *p* = .006, *η*^2^_p_ = .123], the interaction was not significant in the short-term memory task [*F*(1,56) = 3.063, *p* = .086, *η*^2^_p_ = .052]. This discrepancy between the two tasks reflects the major impact of the countermeasures instructions in the detection task but not in the short-term memory task.

Finally, the ANOVA also yielded a significant two-way interaction between familiarity and task [*F*(1,115) = 105.409, *p* < .001, *η*^2^_p_ = .478]. The difference between familiar and unfamiliar faces was significant in both tasks (detection task: *t*(60) = 4.816, *p* < 0.001, *d* = 0.66, short-term memory task: *t*(32) = − 8.766, *p* < .001, *d* = − 2.06). However, the direction of the effect was inversed, in the detection task familiar faces were fixated more across the whole display time (regardless of phase), in the short-term memory task the unfamiliar faces received more fixation time due to the strong preference towards the unfamiliar faces.

### Direct comparison between studies – single display

In contrast to the short-term memory task, in the dot-detection study we did not find a significant difference between the mean fixation duration on familiar and unfamiliar faces in the single display. In order to further explore this finding, we conducted a three-way ANOVA on the mean fixation duration during the single display, with one within-subject factor (familiarity: familiar vs. unfamiliar) and two between-subject factors (type of task in the different studies: short-term memory task vs. visual detection task and experiment instructions: concealed vs. countermeasures). This analysis yielded a main effect of familiarity, reflecting the overall tendency for longer fixations on familiar than on unfamiliar faces [*F*(1,115) = 20.077, *p* < .001, *η*^2^_p_ = .149]. However, there was also a significant interaction between familiarity and task [*F*(1,115) = 4.209, *p* = .042, *η*^2^_p_ = .035], indicating a different modulation of fixation duration in each task. Indeed, a further comparison between familiar and unfamiliar faces across the different tasks demonstrated a strong effect of familiarity in the short-term memory task (*t*(57) = 4.408, *p* < .001, *d* = 0.31), but not in the detection task (*t*(60) = 1.827, *p* = .073, *d* = 0.09).

### ROC analysis

For each experiment, the area under the curve was calculated based on the mean standardized score of the measures described above. As a ROC area of 0.5 signifies chance-level differentiation between trials including familiar faces and trials without any familiar face, the areas obtained were compared to 0.5 using one- sample *t* test. For the concealed experiment, the area under the ROC curve yielded a significantly greater than chance result of (*a* = .807, *p* < .001, CI [.694, .920]). However, in the countermeasures experiment the area did not significantly differed from chance (*a* = .571, *p* = .359, CI [.420, .723])^3^. To compare the detection efficiency of the current study and the previous short-term memory study, we directly compared the area under the curve in the two experiments. The difference between the concealed instructions in the short-term memory task and the dot task was not significant (dot task area under the curve: *a* = .807; short-term memory task area under the curve = .889; *z* = 1.11, *p* = 0.134), but the difference between the tasks in the countermeasures experiment was significant (dot task area under the curve: *a* = .571; short-term memory task area under the curve: *a* = .957; z = 4.79, *p* < .001), reflecting a better detection ability in the countermeasures condition under the short-term memory task.

## Discussion

In two experiments participants performed a visual detection task, in which they saw displays of faces (some familiar and some unfamiliar) and were asked to detect a dot that randomly appeared on one of them. A sole difference distinguished between the two experiments: while in the concealed experiment participants were generally asked to conceal the faces that were familiar to them, in the countermeasures experiment they received precise instructions regarding how to do so (i.e., deploy gaze equally to all faces). This distinction between the two experiments elicited a significantly different gaze behavior; while in the concealed experiment participants preferentially directed their gaze towards familiar faces, this pattern of behavior was reduced in the countermeasures experiment. This discrepancy between the two experiments was evident also when parceling the overall gaze time to smaller units of fixations and visits. This analysis showed that the preference towards familiar faces in the concealed experiment is related to an increased number of fixations and visits, as well as to their increased average duration. In contrast, no significant differences between familiar and unfamiliar faces were found for fixations and visits in the countermeasures experiment.

This pattern of results differed from those reported by Lancry-Dayan et al. (2018) in two main ways. First, in Lancry-Dayan et al. (2018; where the sole difference from the current experiments was the use of a short-term memory task instead of the visual detection task), gaze behavior towards the familiar face was characterized by a short preference followed by a long avoidance. As the visual input in both tasks was similar (i.e., a parallel display of four faces) this discrepancy between the two studies highlights the importance of task demands when considering the interplay between familiarity and gaze preferences. Importantly, the difference between the two studies was not coincidental, but rather reflected the hypothesized theoretical principles of each task; in the short-term memory task, where encoding of the faces is required, a beneficial strategy is to look more on unfamiliar faces. In contrast, in the detection task where no encoding of the faces was needed, the expected orienting response was manifested by preferentially looking at the familiar faces.

These findings expand the work of Ryan et al. (2007) who showed that task instructions determined the direction of gaze towards familiar and unfamiliar faces. In accordance with the results of our previous study (Lancry-Dayan et al., 2018), Ryan et al. (2007) showed that when participants were told to study an array of faces, gaze was drawn towards the unfamiliar ones. While Ryan et al. (2007) considered one of their tasks as “free-view,” we claim that this task may not be “free” at all; participants were encouraged to look more on unfamiliar faces because they require more processing resources (Jackson & Raymond, 2008). In the current study we employed a new method to examine “free viewing” of familiar and unfamiliar faces; by asking participants to detect a dot that appears a few seconds *after* the display onset, we gain a few seconds of “clean” gaze scanning behavior. This task design ensures that participants will have motivation to scan the faces with no need to process them deeply and memorize them. Accordingly, this task uncovers gaze behavior during visual exploration of the faces and demonstrates the hypothesized attraction towards the familiar ones. By tying these findings together with previous ones, the complex interaction between task demands, familiarity and gaze behavior becomes clearer: while preference towards familiar faces is evident during free visual exploration (the current study) and during recognition tasks (Ryan et al., 2007; Schwedes & Wentura, 2012), a preference towards unfamiliar faces is evident during tasks that require encoding of the faces into memory (Lancry-Dayan et al., 2018; Ryan et al., 2007). As our daily life is composed of different tasks, considering the task is essential for providing a more ecological and richer theoretical framework regarding attentional preference in general and its manifestation through eye movements in particular.

The second difference between the current study and the previous one (Lancry-Dayan et al., 2018) is the influence of the countermeasures instructions. While the preference to unfamiliar faces in the short-term memory task (presumably due to easier encoding of familiar faces) has been evident even when participants were instructed to deploy gaze equally, the preference towards the familiar faces has vanished in the countermeasures condition of both tasks. Thus, the difference between the two studies highlights the influence of task demands on the ability to voluntarily control the manifestation of preference in gaze behavior. Specifically, our studies suggest that it is more difficult to intentionally modify gaze behavior patterns when these patterns are crucial for the successful accomplishment of the task. These findings correspond to a larger research field regarding the metacognitive skills of gaze behavior. Studies in this field mainly focused on control of saccades towards salient objects. For example, in the “anti-saccade” task, participants are required to direct their gaze in an opposite direction to where a cue is displayed. For a successful performance, the participants must process the cue, suppress a reflex-like saccade towards it, and then voluntarily generate a saccade toward the opposite side. These studies demonstrated that participants have only partial control over the attraction of gaze towards the cue (Hallett, 1978; Munoz & Everling, 2004). Such partial control was evident also when the salient object was determined by orientation (van Zoest, Donk, & Theeuwes, 2004), abrupt onset (Theeuwes, Kramer, Hahn, & Irwin, 1998), reward value (Bucker, Belopolsky, & Theeuwes, 2015) and even task-irrelevant gaze cues (Kuhn & Kingstone, 2009). However, all these studies used a simple task in which participants were only instructed to prepare a saccade in the direction (or the opposite direction in the case of the anti-saccade task) of the target. Beyond its low ecological validity, this task fails to capture how voluntarily control interacts with task demands and how it unfolds over longer display periods. Our findings indicate that both of these factors should be taken into account when considering intentional control of gaze behavior, as the ability to modify the deployment of gaze changed between the two tasks and over time.

The results we obtained in the countermeasure conditions of both the current and our previous study (Lancry-Dayan et al., 2018) show that the preference toward familiar items could be controlled. This finding may raise doubts regarding our interpretation of the initial preference towards the familiar faces in terms of orienting response. However, a review of the orienting response literature reveals that various theoreticians argued for two types of orienting responses. Specifically, Maltzman and his colleagues (e.g., Maltzman, 1977; Maltzman, Vincent, & Wolff, 1982) made a distinction between voluntary and involuntary orienting responses: the latter is evoked by an unexpected novel stimulus, whereas the former reflects a response to a predictable significant stimulus – a stimulus for which expectations have been formed through instructions. A similar distinction was made by Naatanen (1979), who noted that Sokolov’s (1963) original theory cannot account for the activation of the orienting response by familiar but significant stimuli. Naatanen proposed using the term “orienting reflex” to describe the involuntary organismic response evoked by novel stimuli and reserving the term “orienting reaction” for longer latency, less automatic orienting responses. Clearly, the familiar faces used in our experiments belong to the voluntary orienting response category because participants expected their appearance and were instructed to conceal their familiarity with these faces. Future studies should examine the relationship between gaze preference and orienting response by simultaneously measuring gaze behavior and skin conductance response that has been demonstrated to reflect orienting response (e.g., klein Selle, Verschuere, Kindt, Meijer, & Ben-Shakhar, 2016; klein Selle, Verschuere, Kindt, Meijer, & Ben-Shakhar, 2017).

Finally, our findings demonstrate that the influence of the task on gaze behavior is not limited to a simultaneous display of the faces, but it is also evident when they are presented serially. While previous studies demonstrated that single familiar faces elicit longer fixation durations than unfamiliar faces (Althoff & Cohen, 1999; Heisz & Shore, 2008; Peth et al., 2013, 2016), we replicated this finding in the short-term memory task used in our previous study (Lancry-Dayan et al., 2018) but not in the current visual detection task. Accordingly, we speculate that the longer fixation durations on familiar faces might reflect a recognition process that relies on retrieval from long-term memory, rather than a mere exposure to familiar faces. The interaction between task and familiarity was also evident in the behavioral detection results during the single display. While we previously observed the expected increase in accuracy for familiar faces in the short-term memory task, the opposite pattern of results was observed in the current dot-detection task. This finding implies that even when the task is orthogonal to the familiarity of the faces, processing familiar faces might distract attention from the task, eventually leading to a poorer dot-detection performance. Although this possibility warrants more research, it gains some support from findings showing distraction by familiar faces in comparison to unfamiliar ones during a digit parity task (Devue & Brédart, 2008). It is worth noting that the accuracy rate in the visual detection task was lower than the accuracy rate in the short-term memory task in our previous study (see Fig. 5), implying that the visual detection task was harder than the short-term memory one. Note that in the visual detection task participants were required to report whether a dot appeared on the right or left side of the screen. As the dot sometimes appeared rather close to the center of the screen, the task was not easy and resulted in a relatively low accuracy rate. The fact that the task was not easy implies that the relatively weak detection accuracy in the visual detection task could not be attributed to lack of engagement of the participants.

The contribution of our studies to the theoretical knowledge regarding the impact of task demands and familiarity on gaze behavior and the ability to voluntarily control it, has also practical implications for developing efficient tools to detect concealed information via eye movements. Specifically, a comparison between the two studies highlights the importance of choosing a task that encourages a strategy, which in turn would elicit differential gaze behavior towards familiar and unfamiliar items. The experimental design adopted by Lancry-Dayan et al. (2018) was a short-term memory task, which utilizes interactions between long-term familiarity and short-term encoding, and therefore, yielded stronger effects and better detection efficiency, as compared to the current study and to previous studies using oculomotor and physiological responses. Moreover, these larger effects were observed even under specific instructions to look at all faces equally (i.e., countermeasures). In contrast, these countermeasures instructions diminished the ability to detect concealed faces in the current visual detection task. Thus, tasks that encourage differential gaze behavior are more resistance to countermeasures. Moreover, it is worth noting one strategy that some participants adopted in the visual detection task – to fixate on the center of the screen and detect the abrupt onset of the dot via extra foveal vision (exclusion of these participants from the analysis does not lead to qualitatively different results – for a more detailed analysis, see Additional file 1). Thus, even when participants try to successfully complete the task, they might use a strategy that will lead to the extinction of eye movements. For further discussion of this phenomenon, please see Additional file 1.

Our study demonstrates how task demands modulate the interaction between gaze behavior and familiarity, but clearly, more research is needed in order to provide a comprehensive framework regarding this interplay between task, familiarity and gaze behavior. Specifically, in the current study we examined how familiarity with *faces* interacts with gaze behavior. Thus, this study is limited to conclusions regarding faces and future research is needed for generalizing these gaze patterns for other types of stimuli, such as objects and scenes. Moreover, together with the previous study, we consider only two types of tasks. Obviously, other tasks are available and might elicit different patterns of results, perhaps leading to new theoretical insights and better classification ability.

As described in the “Introduction” section, previous studies demonstrated the manifestation of the orienting response towards familiar items through physiological measures (Ben-Shakhar & Gati, 1987; Gati & Ben-Shakhar, 1990). Although the results of the concealed experiment of both the current and our previous study demonstrated the presumed manifestation of orienting response through eye movements, the results of the countermeasures experiments shed doubts on the interpretation of gaze attraction to familiar faces in terms of orienting response. As advances in technology enable relatively easy recording of gaze behavior simultaneously with physiological responses, future studies should examine gaze patterns together with physiological responses and study whether gaze attraction to significant stimuli is correlated with physiological indices of the orienting response. Beyond clarifying the theoretical insights regarding gaze preference, familiarity and physiological responses, these future studies will be beneficial for converting these insights into efficient applicative tools: first, by providing new paradigms to detect concealed information with other types of stimuli, and second by fine-tuning the task in order to lead to large differences between familiar and unfamiliar items, enabling better classification ability.

## Conclusions

In a series of experiments, we established the importance of the task in the interplay between gaze preference and familiarity as well as for applicative tools for the detection of concealed information via eye movements. By comparing the short-term memory task (used in our previous study) to the current dot-detection task, we showed how different theoretical insights are manifested via eye movements and demonstrated that tasks for detecting concealed memories should be tailored according to a solid theoretical background. Currently, the short-term memory task seems to be an excellent candidate for detecting concealed information via eye movements, as it elicits impressive classification rates and shows promising results even under countermeasure efforts. Thus, the use of eye tracking during encoding of parallel displays into short-term memory proves to be very beneficial in utilizing theoretical knowledge in applied detection of familiarity. We are hopeful that this line of research will continue to expand the theoretical insights on memory-guided attention alongside its applied implications.

## Additional file

Additional file 1: Detecting concealed familiarity using eye movements: the role of task demands. (DOCX 30 kb)

## Abbreviation

CIT: Concealed information test

## Footnotes

1 In order to compare the results with those reported by Lancry-Dayan et al. (2018), we kept the same parameters. Thus, although we did not anticipate two phases of attention in this experiment, we divided the dwell time phases before the dot appeared into two periods: 0–1000 ms and 1000–3000 ms, and did not include the visit duration measure that was not used in Lancry-Dayan et al. (2018).

2 As in the analysis of the previous study (Lancry-Dayan et al., 2018), we did not include the accuracy scores in the ROC because it is a binary variable which we could not standardize.

3 We performed another classification method using a support vector machine analysis that classified all the pictures as unfamiliar. See the Additional file 1 for details regarding the SVM classification.
