# Supplementary material for: Detecting concealed familiarity using eye movements: the role of task demands
Source: Cogn Res Princ Implic. 2019 Mar 29;4:10. doi: 10.1186/s41235-019-0162-7 (PMC6439126; doi:10.1186/s41235-019-0162-7)
Supplement: Supplementary file 1 — Detecting concealed familiarity using eye movements: the role of task demands. (DOCX 30 kb) [file 41235_2019_162_MOESM1_ESM.docx]

**Detecting concealed familiarity using eye movements: the role of task demands**

**Supplementary material**

**Summary of the Bayesian analyses.**

In both experiments, for each of the measures we built the same model, and used different priors based on the pooled standard deviations as described in the paper (see below):

model{

for (i in 1:ndata){

diff[i] ~ dnorm(mu ,lambda)}

mu <- delta*sigma

lambda <- 1/sigma^2

lambdadelta ~ dchisqr(1)

delta ~ dnorm(0,lambdadelta)

sigma ~ dunif(0, sd_diff * 10)}

|  | | **concealed** | **countermeasures** |
| --- | --- | --- | --- |
|  | | **Standard deviation pooled** | **Standard deviation pooled** |
| **Parallel display** | **Number of fixations** | 0.39 | 0.39 |
|  | **Duration of fixations** | 24.45 | 27.9 |
|  | **Number of visits** | 0.17 | 0.19 |
|  | **Duration of visits** | 62.3 | 77.85 |
| **Single display** | **Duration of fixations** | 39.84 | 73.12 |
|  | **Accuracy** | 0.06 | 0.07 |
|  | **Reaction time** | 73.2 | 85.92 |

**Summary of the visit analysis adjusted to the previous experiment.**

The analysis of visits that was carried out in our previous study (Lancry-Dayan, Nahari, Ben-Shakhar, & Pertzov, 2018) is slightly different from the one we conducted in the current study. While in the current study we examined the overall mean number of visits, in the previous study we examined the proportion of trials in which one, two, three (or more) visits were executed towards familiar and unfamiliar faces. Moreover, in the previous study we did not report the mean duration of the visits. We believe that the current approach is simpler and clearer. Accordingly, we report the overall difference between familiar and unfamiliar faces regarding the number of visits and their duration in the main text. To enable a direct comparison with the visit analysis of the previous study, below we adopted the analysis method used in our previous study and report the results separately for each visit (see below).

Concealed experiment: There was no difference in the proportion of trials in which only two visits were made towards familiar and unfamiliar faces (t(32) = .391, p =.705, d = 0.06). However, unfamiliar faces were visited only once in more trials than familiar faces (t(32) = 2.319, p = .027, d = 0.4). Moreover, familiar faces were visited three (or more) times in more trials than unfamiliar faces (t(32) = 3.804, p = .001, d = 0.66). Thus, the preference effect may emerge from a tendency to visit the familiar face more. The preference effect may be also due to longer visits on familiar faces. Indeed, we found a significant difference in the mean duration of the first visit (t(32) = 5.836, p < .001, d = 1.02) and a marginally significant difference for the second visit (t(32) = 1.9, p = .066, d = 0.33). There was no significant difference in the duration of the third visit (t(28*) = .331, p = .743, d = 0.06).

Countermeasures experiment: In this experiment, only half of the participants had more than two visits for both familiar and unfamiliar faces, thus we compared only the first and second visits. Overall, there was no significant difference between familiar and unfamiliar faces regarding the frequency of trials with one visit (t(27) = .349, p = .730, d = 0.06) or two visits (t(27) = .057, p = .955, d = 0.01). However, the first visits on the familiar faces were significantly longer (t(27) = 2.234, p = .034, d = 0.42), but no significant difference was found for the duration of the second visit on familiar and unfamiliar faces (t(26*) = 1.139, p = .265, d = 0.22).

* Some participants did not have more than one or two visits, thus, leading to fewer degrees of freedom in the duration analysis (these participants were included as zero for the proportion analysis).

**Table S1** The four-way ANOVA results, comparing between the short-term memory task and the visual detection task.

In order to compare the effects between the two studies, we conducted a 4- way ANOVA on the dwell time during the parallel display, with two within-subjects factors (familiarity: familiar vs. unfamiliar and dwell time phase: first vs. second) and two between-subjects factors (type of task: short-term memory task vs. visual detection task and experiment: concealed vs. countermeasures). In the main text we discuss only the effects that include the factors of familiarity (three way interactions and below). Here, we provide the overall results of this analysis, followed by a short discussion of the significant ones (three way interactions and below).

|  | F | Degrees of freedom | p | η^2^_p_ |
| --- | --- | --- | --- | --- |
| Dwell time phase | 7462.269 | 1,115 | <.001 | .985 |
| Familiarity | 42.395 | 1,115 | <.001 | .269 |
| Dwell time phase * familiarity | 65.111 | 1,115 | <.001 | .362 |
| Dwell time phase * task | 1318.827 | 1, 115 | <.001 | .920 |
| Dwell time phase * experiment | 4.129 | 1, 115 | .044 | .035 |
| Familiarity * experiment | .282 | 1,115 | .596 | .002 |
| Familiarity * task | 105.409 | 1,115 | <.001 | .478 |
| Dwell time phase * familiarity * task | 92.065 | 1,115 | <.001 | .445 |
| Dwell time phase * familiarity * experiment | 2.193 | 1,115 | .141 | .019 |
| Dwell time phase * experiment * task | 2.656 | 1,115 | .106 | .023 |
| Familiarity * task * experiment | 7.615 | 1,115 | .007 | .062 |
| Dwell time phase * familiarity * task * experiment | 5.265 | 1,115 | .024 | .044 |

As the second dwell time was longer than the first one, it is not surprising that we found a strong effect for the dwell time phase. To further understand the significant two way interactions of dwell time phase with the other factors we conducted three additional t-tests. First, in order to understand the interaction between dwell time phase and task, we conducted a paired t-test comparing between the two phases, separately for each experiment. This analysis yielded a significant difference between the two phases for both the short-term memory task (t(57) = 74.714, p<.001, d = 9.81) and the detection task (t(60 = 40.808, p<.001, d =5.22)), thus indicating that the significant interaction is due to the stronger effect in the short-term memory task. A similar pattern emerged also when investigating the interaction between dwell time phase and condition. The paired t-test showed that the difference between the first phase and the second phase was significant in both the concealed experiment (t(61) = 17.571, p<.001, d = 2.23) and the countermeasures experiment (t(56) = 17.027, p<.001, d = 2.25). Finally, we examined the significant interaction between dwell time phase and familiarity. A paired t-test that compared between the first and second phase, separately for familiar and unfamiliar faces, showed a significant difference in dwell time during the first and second phases for both familiar (t(118) = 22.670, p<.001, d = 2.07) and unfamiliar faces (t(118) = 19.719, p<.001, d = 1.81). Accordingly, once again the significant interaction is due to difference in the effect size. Beyond the effect of dwell time, familiarity was also a significant factor in this model, reflecting an overall tendency to look less on familiar faces, probably due to the strong avoidance effect in the short-term memory task.

**Summary of the support vector machine (SVM) analysis.**

We used the SVM supervised learning algorithm (Shalev-Shwartz & Ben-David, 2014) with the four gaze features derived from the parallel display (similarly to Lancry-Dayan et al., 2018): dwell time on the first and second time phases, number of fixation and number of visits. For each participant we averaged each eye tracking measure across the four presentations of the picture and standardized the scores across all pictures. This procedure yielded a dataset of all participants including the standardized four measures for each picture together with their tag as familiar or unfamiliar. Given a subset of these data as training, the SVM classified a new set of pictures as familiar versus unfamiliar. The classification rate refers to the ability of the SVM to correctly classify a picture from the test set as familiar or unfamiliar. We conducted two classification types – a *within-subject* classification (for each participant, training on the data of 75% of the trials and testing on the data of the rest 25% of the trials) and a *between-subject* classification (for each participant, training on all other participants and testing on the data of that participant).

Results:

***Within subject analysis****.* The SVM was trained on three folds of each participant’s pictures (48 pictures), and then attempted to classify the remaining fold (16 pictures) as familiar or unfamiliar, with the same proportion of familiar and unfamiliar pictures as the original data. This process was iterated four times, each time training and testing on a different combination of the four folds, thus ensuring that each picture would be included once in the test fold. The average classification score for these four iterations within each participant was then aggregated across all participants. Unlike the results reported by Lancry-Dayan et al. (2018), the current analysis revealed that all pictures were classified as unfamiliar, thus, indicating that the eyes measures did not provide efficient information for the picture classification.

***Across subjects analysis.*** This SVM aimed to classify correctly the familiar pictures of one participant, based on the training data consisting of all other participants but one. This process was iterated for each participant, each time training on the datasets of all the other participants and testing classification accuracy on the data of the remaining one. Once again, all pictures were classified as unfamiliar.

**Summary of the concealed experiment analysis, excluding problematic participants.**

During the concealed experiment, two participants adopted a remarkable strategy: instead of actively searching for the dot, they fixated on the center of the screen and detected the abrupt onset of the dot. Participants were identified as using this strategy if during the time before the dot onset more than 5 consecutive of their trials did not include fixations at all. Moreover, we confirmed that the participants used this strategy by checking some of the videos of their problematic trials. These videos verified that these participants looked at the center of the screen for the whole period before the dot appeared. Two participants met this criterion and were identified as using this strategy in the concealed experiment (first participant: 29 trials without any fixations, second participant: 22 trials without any fixations), but none in the countermeasures experiment. Here we provide a brief discussion of the main eye movements results of the concealed experiment without these two participants, indicating that their exclusion did not change the overall pattern of results in this experiment.

The exclusion of the two participants from the analysis did not change the preference towards the familiar face. Accordingly, participants looked more on the familiar face, starting around 500 ms after display onset until about 2200 ms (significant below α<.05 (except of a small gap of 200 ms), after false discovery rate (FDR) correction for multiple comparisons). Beyond the overall gaze time, also the effects of visits and fixations reminded the same: participants visited familiar faces more (t(30) = 3.292, p =.003, d = 0.59), each visit lasting longer (t(30) = 5.026, p<.001, d = 0.9). Moreover, they directed more fixations (t(30) = 5.154, p <.001, d = 0.49) and longer ones (t(30) = 2.754, p =.01, d = 0.92) towards familiar faces. Finally, also the findings regarding fixations duration during the single display showed a similar pattern: there was still no significant difference between familiar and unfamiliar faces (t (30) = 1.558, p = .130, d = 0.28).
